# Two new species of *Zospeum* Bourguignat, 1856 from the Basque-Cantabrian Mountains, Northern Spain (Eupulmonata, Ellobioidea, Carychiidae)

**DOI:** 10.3897/zookeys.483.9167

**Published:** 2015-02-23

**Authors:** Adrienne Jochum, Anton J. de Winter, Alexander M. Weigand, Benjamín Gómez, Carlos Prieto

**Affiliations:** 1 Naturhistorisches Museum der Burgergemeinde Bern, CH-3005 Bern, Switzerland; Institute of Ecology and Evolution, University of Bern, CH-3012 Bern, Switzerland; 2 Naturalis Biodiversity Center, P.O. Box 9517, 2300 RA Leiden, The Netherlands; 3 Department of Animal Ecology, Evolution and Biodiversity, Ruhr University Bochum, 44801 Bochum, Germany; 4 Department of Zoology, University of the Basque Country (UPV/EHU), 01006-Vitoria, Spain; 5 Department of Zoology and Animal Cell Biology, Faculty of Science and Technology, University of the Basque Country (UPV/EHU), 48080-Bilbao, Spain

**Keywords:** Cave-dwelling species, subterranean snail, microgastropoda, pseudo-cryptic species, shell variability, ecology, conservation

## Abstract

Two new species of the genus *Zospeum* Bourguignat, 1856 are described from caves in the Sierra de Aitzgorri (Gipuzkoa) and the Sierra Salvada (Burgos) in Northern Spain. The taxa *Zospeum vasconicum* **sp. n.** and *Zospeum zaldivarae* **sp. n.** have recently, without a formal name, been included in a molecular study of worldwide members of the Carychiidae. In the present paper, the shell morphology and variation of these species is described and illustrated.

## Introduction

The subterranean genus *Zospeum* Bourguignat, 1856 (Ellobioidea, Carychiidae) encompasses a Palearctic radiation of terrestrial snails. These unpigmented, blind gastropods are amongst the smallest terrestrial gastropods known, with some species barely reaching 1 mm in shell size and inhabiting caves at depths as deep as 950 m (Weigand 2013). *Zospeum* species inhabit moist, muddy cave walls, rock crevices, speleothems and ceilings in the deep recesses of karst caves, far from the entrance zone. Stable environmental conditions such as minimal fluctuations in temperature, humidity, airflow, water levels and the constant influx of organic matter are characteristic for caves harbouring *Zospeum* populations throughout their known Cantabrian, Pyrenean, Southern Alpine and Dinaric distributions. The greatest species diversity is recorded from caves located in the vast karst regions of south-central and south-eastern Europe (see Bole 1974, Pezzoli 1992). The past four decades have witnessed a number of species discoveries in northern Spain, viz., *Zospeum bellesi* Gittenberger, 1973, *Zospeum suarezi* Gittenberger, 1980, *Zospeum biscaiense* Gómez & Prieto, 1983 and Jochum et al. 2012, whereby the oldest Iberian record dates back to the mid 19^th^ century (*Zospeum schaufussi*, von Frauenfeld, 1862). This latter description was long overlooked until first quoted by Giusti (1975). A faunistic overview of the land snails of northern Spain (Altonaga et al. 1994) indicated the existence of four more species (*Zospeum* sp. n. 1–4). So far, these taxa have not been described.

Additional collecting in northern Spain by various workers has yielded extensive new *Zospeum* material, revealing the existence of yet still more taxa. This study is ongoing. In many cases, the considerable variability observed between and within different cave systems precludes easy delimitation of species using shell morphology alone.

In a recent paper, Weigand et al. (2013) addressed the evolution of the worldwide members of the entire family Carychiidae, including a number of Iberian *Zospeum* species. Two of these remain unnamed, yet proved to be molecularly distinct from all Iberian (and other) taxa studied thus far. In order to augment our knowledge of biodiversity in general, and specifically here for Spain, as well as to deposit their DNA sequences in public databases such as GenBank, these taxa will be described and their shell morphology compared to Iberian taxa already known. This concerns the two molecularly flagged, but so far unnamed lineages Z17 and Z18 (morphospecies *Zospeum* sp. 1 and *Zospeum* sp. 2) in Weigand et al. (2013).

## Material and methods

**Figure 1.**
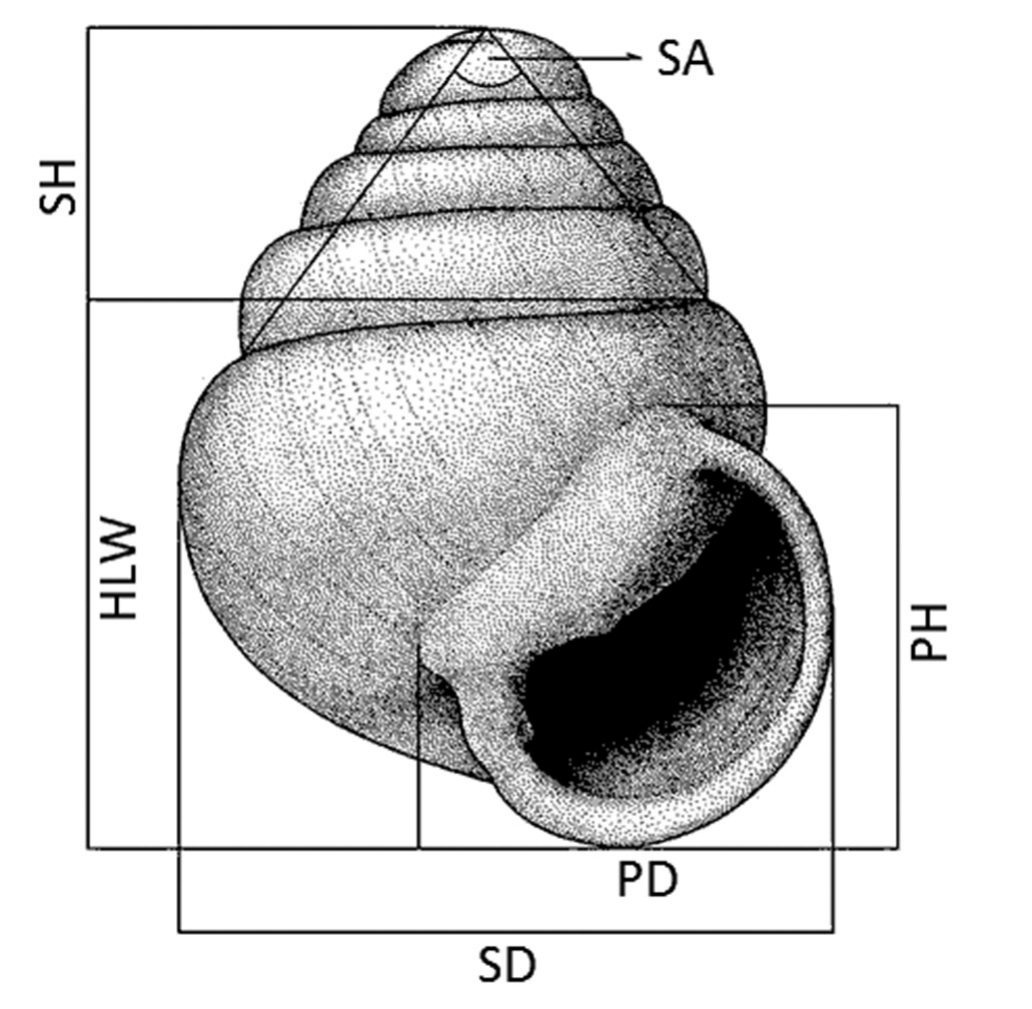
Measurement on *Zospeum* shells in frontal view. Abbreviations: **HLW** height of last whorl **PH** peristome height **PD** peristome diameter **SA** spire angle **SD** shell diameter **SH** shell height.

Shells were measured as indicated in Figure 1. The number of whorls of each shell was counted according to the method described in Kerney and Cameron (1979). For the species descriptions, shell measurements are expressed as ratios such as SH:SD, HLW:SH, PH:SH, PH:PD, W:*ln*H (coiling tightness, Emberton 2001). Measurements were taken from images obtained by either a Leica DFC420 digital camera attached to a Leica M165c stereo microscope, using Leica LAS V4.4 software; or with a Nikon digital camera attached to a Nikon SMZ1500 stereo microscope, using the Nikon DS-L1 analysis image system software for measurements.

We also address a number of qualitative aspects of shell morphology: peristome shape; whorl profile (whorl convexity); regularity of the protoconch; teleoconch sculpture; development of apertural barriers visible in frontal view, including the presence/absence of a deeply immersed denticle/lamella on the parieto-columellar region of the aperture; development of the columellar lamella as discernable in very fresh, transparent shells, or by perforating the body whorl of the adult shell.

Material is housed in the following collections:

AJC Adrienne Jochum Collection: formerly Institute of Ecology, Evolution & Diversity, Phylogeny & Systematics Collection, Goethe-Universität, Frankfurt am Main, Germany

MCBI CSR SASA Malacological collection of the Biological Institute of the Centre for Scientific Research of the Slovenian Academy of Sciences and Arts in Ljubljana, Slovenia

MNCN Museo Nacional de Ciencias Naturales, Madrid, Spain

NMBE Naturhistorisches Museum der Burgergemeinde Bern, Bern, Switzerland

RMNH Naturalis Biodiversity Center (formerly RijksMuseum van Natuurlijke Historie), Leiden, The Netherlands

SMF Forschungsinstitut und Naturmuseum Senckenberg, Frankfurt am Main, Germany

UPV/EHU-FC Colección de Fauna Cavernícola (Departamento de Zoología) de la Universidad del País Vasco-Euskal Herriko Unibertsitatea, Bilbao, Spain

## Taxonomy

### Family Carychiidae Jeffreys, 1830 Genus *Zospeum* Bourguignat, 1856

#### Zospeum vasconicum

Taxon classificationAnimaliaPulmonataEllobiidae

Prieto, De Winter, Weigand, Gómez & Jochum sp. n.

http://zoobank.org/90EF1F13-9D16-4F08-9398-DCF7CFDE17AA

Figures 3 , 4 , 5

Zospeum sp. n. 1, Altonaga et al. 1994: 72 (in part).
Zospeum sp. n. 1, Jochum et al. 2012: 402, Fig. 3 A.
Zospeum sp. n. 1, Weigand et al. 2013: 8, Fig. 2.

##### Material.

*Type material.* Holotype (MNCN15.05/60147H): Spain, Prov. Gipuzkoa, Oñate,Valle de Araotz, Cueva de la Ermita de Sandaili, UTM 30TWN4580260906, N42.999442, E-2.438076, alt. c. 400 m, moist, muddy walls in karst cave, 15.11.1984, leg. C. Prieto, B. Gómez & K. Altonaga.

Paratypes: locus typicus: 53 shells (UPV/EHU-FC: 74) and 4 dried snails (UPV/EHU-FC: 75), data as the holotype; 41 shells (UPV/EHU-FC: 549), 18.06.2011, leg. C. Prieto, A. Jochum, A. Weigand, R. Slapnik & J. Valentinčič; 6 shells (MNCN15.05/60147P, ex UPV/EHU-FC: 549), ibid.; 6 shells (SMF 341634, ibid.), ibid.; 6 shells (RMNH.5003914, ibid.), ibid.; 6 shells (NMBE 529864/6, ibid), ibid.; 19 shells (AJC/1864), ibid.

**Figure 2.**
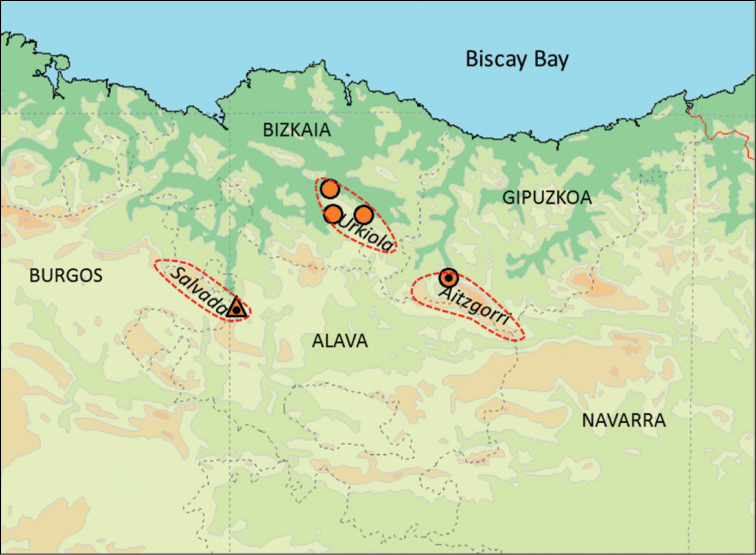
Map indicating geographic position of massifs and caves with *Zospeum* species. Dot in Triangle, *Zospeum zaldivarae* sp. n.: Cueva de Las Paúles (locus typicus); Dot in circle, *Zospeum vasconicum* sp. n.: Cueva de la Ermita de Sandaili (locus typicus); Simple orange circles, *Zospeum vasconicum* sp. n. localities: Cueva de Otxas; Cueva Silibranka-2; Cueva del Cranéo.

*Other material.* (Fig. 2): Prov. Bizkaia: Yurre, Urkizu, Cueva de Otxas, UTM 30TWN2050081208, N43.183362, E-2.747741, alt. c. 500 m, 18.01.1981, leg. B. Gómez, R. Martín, K. Altonaga, 23 shells (UPV/EHU-FC:24); same locality, 19.06.2011, leg. C. Prieto, A. Jochum, A. Weigand, R. Slapnik & J. Valentinčič, 30 shells (MCBI CSR SASA 40115), ibid., 20 shells (AJC/1867), ibid. 11 shells (RMNH.5003916); Mañaria, Cueva de Silibranka-2, UTM 30TWN2741175235, N 43.129357 E -2.662995, alt. 220m, 20.06.2011, leg. C. Prieto, A. Jochum, A. Weigand, R. Slapnik & J. Valentinčič, 80 shells (UPV/EHU-FC:557), ibid., 5 shells (MCBI CSR SASA 40090), ibid., 18 shells (AJC/1851), ibid. 10 shells (RMNH.5003915); Dima, Indusi, Cueva del Cráneo, UTM 30TWN2157275145, N43.128736, E-2.734786, alt. c. 400 m, 20.06.2011, leg. C. Prieto, A. Jochum, A. Weigand, R. Slapnik & J. Valentinčič, 13 shells (UPV/EHU-FC:556), ibid., 13 shells (AJC/1853).

##### Diagnosis.

Shell *ca.* 1.2 mm, transparent, elongate or elongate-conical with an entire, roundish and more or less thickened peristome, lacking obvious apertural barriers, but often with an obsolete lamella (denticle) in the parieto-columellar corner; columella with a single, low annular lamella.

##### Description

(material from type locality). Measurements of holotype and paratypes are provided in Table 1.

**Table 1.** Holotype dimensions, and mean, maximum (max), minimum (min), and standard deviation (sd) of shell measurements (see Figure 1) of four populations of *Zospeum vasconicum* sp. n.: Cueva de la Ermita de Sandaili, N=20; Cueva de Otxas, N=11; Cueva Silibranka-2, N=10; Cueva del Cranéo, N=11. SH – shell height, SW – shell width, HLW – height of last whorl, PH – peristome height, PD – peristome diameter, SA – spire angle, W – number of whorls, CT– coiling tightness. SA in degrees, other measurements in mm.

Sandaili	SH	SW	HLW	PH	PD	SA	W	H/D	HLW/H	PH/H	CT	PH/PW
**holotype**	1.20	0.83	0.77	0.43	0.43	59	4.7	1.45	0.64	0.36	25.78	1.0
mean	1.23	0.84	0.76	0.46	0.46	58	4.95	1.47	0.62	0.37	25.59	1.0
max	1.45	0.92	0.85	0.51	0.53	65	5.5	1.58	0.65	0.41	42.35	1.11
min	1.12	0.77	0.67	0.4	0.4	52	4.6	1.34	0.55	0.34	14.80	0.87
sd	0.07	0.046	0.041	0.035	0.030	3.68	0.228	0.072	0.027	0.023	6.31	0.068
**Silibranka**												
mean	1.29	0.81	0.73	0.45	0.43	56.3	5.55	1.59	0.57	0.35	22.42	1.04
max	1.38	0.86	0.76	0.51	0.46	60	6	1.71	0.58	0.38	27.10	1.13
min	1.23	0.75	0.67	0.41	0.40	49	5.25	1.52	0.54	0.33	18.71	0.99
sd	0.048	0.033	0.029	0.029	0.018	3.335	0.194	0.054	0.016	0.016	3.068	0.044
**Otxas**												
mean	1.29	0.80	0.74	0.44	0.45	58.5	5.29	1.61	0.58	0.34	21.04	0.98
max	1.36	0.85	0.82	0.48	0.49	65	5.6	1.70	0.60	0.37	24.44	1.05
min	1.24	0.74	0.68	0.40	0.42	54	5	1.46	0.54	0.32	17.44	0.933
sd	0.046	0.037	0.038	0.025	0.024	3.725	0.194	0.078	0.024	0.017	2.608	0.032
**Cranéo**												
mean	1.24	0.87	0.80	0.52	0.50	63.1	4.97	1.41	0.64	0.42	23.59	1.05
max	1.28	0.9	0.88	0.62	0.55	68	5	1.48	0.69	0.48	26.23	1.15
min	1.21	0.84	0.75	0.44	0.46	61	4.85	1.38	0.61	0.35	20.25	0.90
sd	0.021	0.018	0.039	0.049	0.027	2.514	0.063	0.032	0.024	0.038	1.742	0.087

Shell minute, rather variable in height (on average *ca.* 1.2 mm), conical to elongate-conical with about 5 whorls, regularly coiled, suture deep, whorls convex, more or less strongly shouldered, especially in the more conical shells; teleoconch sculpture of fine, occasionally almost rib-like, axial striae; weak axial ribbing immediately behind the palatal-basal lip, occurring for a short distance; aperture more or less circular; peristome closely adhering to spire, reflected, moderately thickened, roundish, but often somewhat higher than wide or wider than high, taking up *ca.* 40% of shell height; umbilicus closed, umbilical depression deep, with fine or coarser, sometimes almost rib-like, axial striae; apertural barriers absent apart from a rather low lamella (appearing as a tiny denticle) on the parietal-columellar corner, discernable only in oblique apertural view; columella with a single, low annular lamella, only visible in body whorl at some distance from aperture.

**Figure 3.**
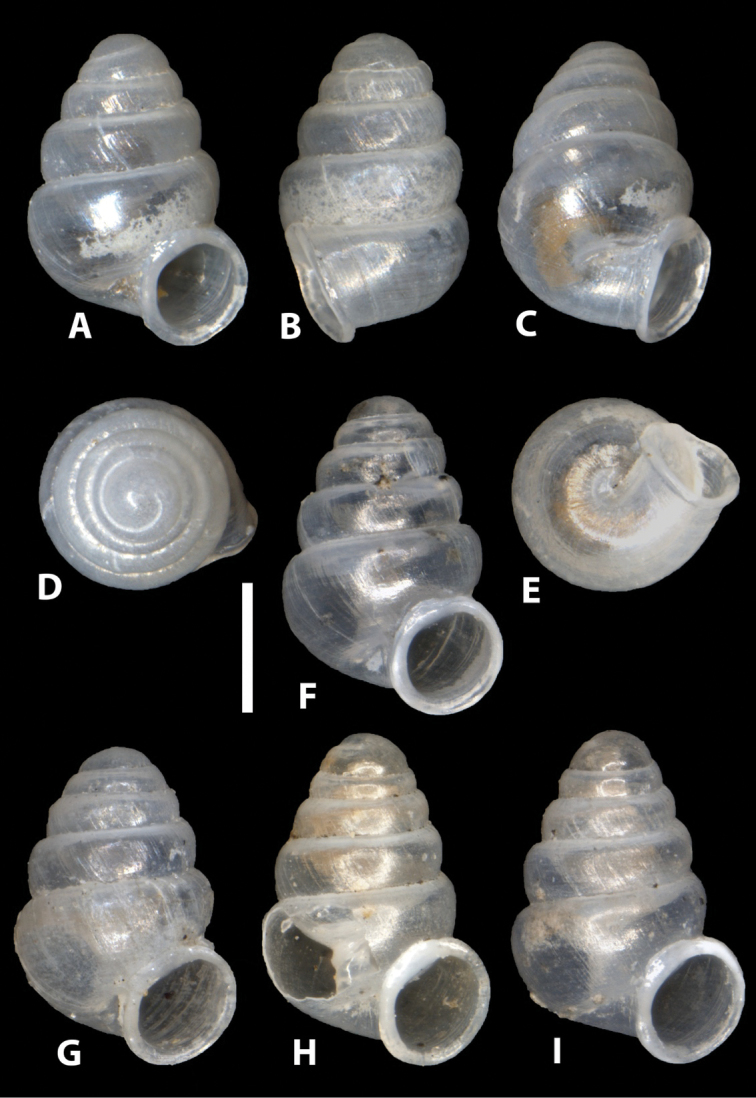
*Zospeum vasconicum* sp. n., **A–E** different views of holotype (MNCN 15.05/60147H) **F–I** paratype shells (MNCN 15.05/60147P) **H** paratype shell with window cut in body whorl exposing columella. Scale bar 0.5 mm.

**Figure 4.**
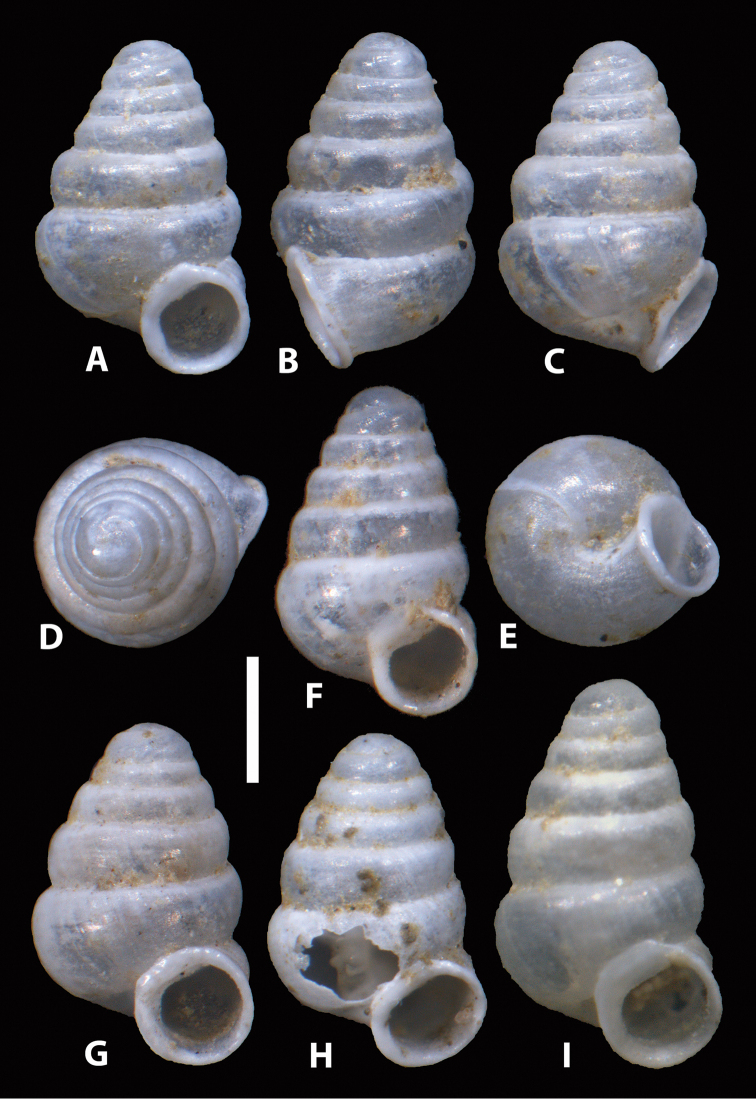
*Zospeum vasconicum* sp. n., **A, F–I** shells from Cueva Silibranka-2 in frontal view (RMNH.5003915) **B–E** different views of specimen **A**; **H** shell with window cut in body whorl exposing columella. Scale bar 0.5 mm.

**Figure 5.**
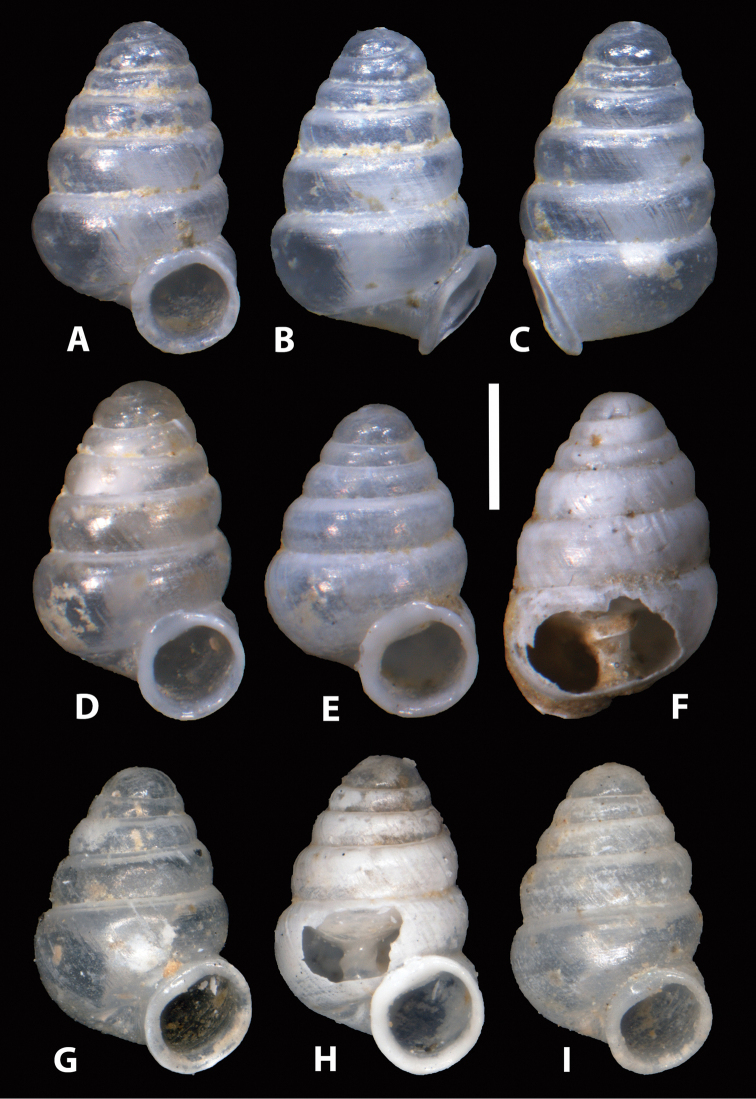
*Zospeum vasconicum* sp. n., Shells from Cueva de Otxas (**A–F** RMNH.5003916); and Cueva del Cranéo (**G–I** UPV/EHU-FC: 556). **B–C** different views of **A**; **F, H** shells with window cut in body whorl exposing columella. Scale bar 0.5 mm.

##### Differential diagnosis.

Differs from *Zospeum biscaiense* by the smaller, more elongate shell and the absence of major apertural barriers; from *Zospeum schaufussi* (*sensu* Gittenberger 1980) by the roundish peristome; *Zospeum suarezi* is generally smaller, and has a more elaborate, two-tiered arrangement of lamellae on the columella; in *Zospeum bellesi*, apertural barriers and columellar ornamentation are completely absent.

##### Etymology.

The new species is named after the pre-Roman Era Vascones Tribe (from Latin *gens Vasconum*), which at the arrival of the Romans during the 1^st^ century, inhabited a territory spanning the region between the upper course of the Ebro River and the southern basin of the western Pyrenees. This tribe is considered (disputed) the ancestor of the Basque People.

##### Distribution.

Sierra de Aitzgorri and the adjacent Sierra de Aramotz-Anboto in the Provinces of Gipuzkoa and Bizkaia, Spain (Fig. 2). Both massifs are formed in Lower Cretaceous (Urgonian) limestone bedrock and separated by the valley excavated by the Deba River.

##### Ecology.

Live *Zospeum vasconicum* were found in Cueva Arrikrutz on densely perforated mats of fine mud lining the walls of the upper level of the cave. In the immediate vicinity of this colony, numerous translucent *Zospeum* shells were found embedded in a thick, uniform layer of mud, superficially interspersed with yellow, clumped strands of fungal aggregations (Fig. 8A–B). Only single live individuals of *Zospeum vasconicum* were found on the walls of Cueva de la Ermita de Sandaili. No bats or bat guano were seen in the vicinity of the collection site.

##### Conservation.

In the caves where this species occurs (see above), fresh empty shells were found in relative abundance at various spots within these caves, suggesting that the species commonly occurs there, and that these populations are not immediately threatened. Still, on a global scale, its distribution is likely limited to less than 5 caves within a radius of less than 20 km^2^. In conjunction with the categories for the IUCN Red List (IUCN Standards and Petitions Subcommittee 2014), it is considered a vulnerable, narrow range endemic (Vu, D2). Habitat disturbance by unrestricted tourism may pose the largest threat. The cave entrance of Cueva de la Ermita de Sandaili contains a chapel, is openly accessible and is not protected as an entity within a natural park. Neighbouring Cueva Arrikrutz belongs to the Natural Park of Aizkorri-Araotz and opened for tourism in June 2007.

##### Remarks.

Although the populations studied in this paper were collected from currently non-contiguous caves, which are geologically part of two adjacent limestone complexes i.e. Otxas, Cráneo and Silibranka-2 of the Aramotz-Anboto massif and Sandaili of the Aizkorri massif, these populations were found to be very closely related, sharing identical or very similar CO1, 16S and H3 sequences (Weigand et al. 2013). However, morphologically, significant differences exist between these populations. Although shell dimensions are quite variable, even within populations, populations differ more or less in shell size and shape (see Table 1). Shells from the type locality and from Cueva del Cráneo, are on average, smaller, significantly less slender, have less whorls and a proportionately larger body whorl than those from Cueva Silibranka-2 and Cueva de Otxas, but the range of some characters overlap. These populations are not different in spire angle and coiling tightness. Shells from Cueva del Cráneo seem to have larger peristomes than specimens from the other caves. Observed are additional qualitative differences between the populations such as in sculptural texture (coarseness of rib-striation; ribbing behind palatal lip present in type locality and Cueva del Cráneo, indistinct or absent in Silibranka-2 and Otxas); whorl profile (topotypic shells have more convex and more strongly shouldered whorls than e.g. the Silibranka-2 population), as well as in the expression of the parieto-columellar denticle (obsolete or absent in Silibranka-2 but generally present in shells from the other caves). In some Cueva de Otxas shells, the peristome is slightly detached from the spire (Fig. 5C). We restrict the type material to (selected) shells from the Cueva de la Ermita de Sandaili. However, material documented by Jochum et al. (2012) from the neighbouring Cueva Arrikrutz within the Natural Park of Aizkorri-Araotz, Oñate (N42.997222, W2.428076), is likely also to be *Zospeum vasconicum*. The nearest passage of the Arrikrutz-Gesaltza cave system is less than 150 m from the Cueva de la Ermita de Sandaili on the other side of the river. However, the Arrikrutz material was not molecularly assessed by Weigand et al. (2013).

#### Zospeum zaldivarae

Taxon classificationAnimaliaPulmonataEllobiidae

Prieto, De Winter, Weigand, Gómez & Jochum sp. n.

http://zoobank.org/8C10D84B-0558-443A-87C7-20E499A3963D

Figures 6 , 7

Zospeum sp., Prieto and Gómez 1985: 145, Fig. 3 A–B.
Zospeum sp. n. 3, Altonaga et al. 1994: 73 (in part).
Zospeum sp. n. 3, Jochum et al. 2012: 402, Fig. 3 B.
Zospeum sp. 2, Weigand et al. 2013: 8, Fig. 2.

##### Material.

*Type material.* Holotype (MNCN15.05/60148H)): Spain, Prov. Burgos, Berberana, Monte de Santiago, Cueva de Las Paúles, UTM 30TWN0062054680, N43.1282, E-2.73618, alt. c. 840 m, moist, muddy walls in karst cave, 09.11.2013, leg. C. Prieto.

Paratypes: locus typicus: 8 specimens (MNCN15.05/60148P ex UPV/EHU-FC:1608) and 2 shells (NMBE 529904/2), 2 shells (SMF 341635) and 2 shells (RMNH.5003943), data as the holotype. 1 shell (UPV/EHU-FC:64), 12.02.1984, leg. P. Zaldívar. 2 shells (MNCN 15.05/60149, ex UPV/EHU-FC:70) and 3 shells (UPV/EHU-FC:72), 11.11.1984, leg. C. Prieto, B. Gómez & P. Zaldívar, 9 specimens (UPV/EHU-FC:559), 21.06.2011, leg. C. Prieto, A. Jochum, A. Weigand, R. Slapnik & J. Valentinčič. 7 specimens + 5 shells (UPV/EHU-FC:560), ibid., 3 specimens molecularly processed (Weigand et al. 2013), 7 shells (4 broken) (AJC/1877), ibid., 5 shells (broken) (MCBI CSR SASA 40598), 11.11.1984, leg. C. Prieto & B. Gómez, 3 shells (RMNH.234152).

##### Diagnosis.

Shell turbinate-conical with approximately 5 ½ regularly coiled, convex, rounded whorls, transparent, comparatively large; columellar and palatal-basal lip narrowly reflected; umbilicus closed, umbilical depression deep.

##### Description.

Measurements are provided in Table 2.

**Table 2.** Holotype dimensions and summary of shell measurements (mean, maximum (max), minimum (min), and standard deviation (sd)) of type material of *Zospeum zaldivarae* sp. n.: SH – shell height, SW – shell width, HLW – height of last whorl, PH – peristome height, PD – peristome diameter, SA – spire angle, W – number of whorls, CT– coiling tightness. SA in degrees, other measurements in mm.

	SH	SW	HLW	PH	PW	SA	W	SH/SW	HLW/SH	PH/SH	CT	PH/PW
**holotype**	1.52	1.25	0.96	0.78	0.73	70	5.4	1.22	0.63	0.51	12.9	1.07
**mean**	1.50	1.16	0.94	0.73	0.69	66.60	5.53	1.29	0.63	0.49	13.90	1.06
**max**	1.66	1.25	1.02	0.80	0.78	75.00	6.20	1.42	0.67	0.52	16.70	1.17
**min**	1.39	1.06	0.83	0.68	0.60	60.00	5.15	1.21	0.58	0.46	11.81	1.00
**sd**	0.070	0.063	0.056	0.041	0.051	3.888	0.268	0.072	0.025	0.020	1.397	0.048

**Figure 6.**
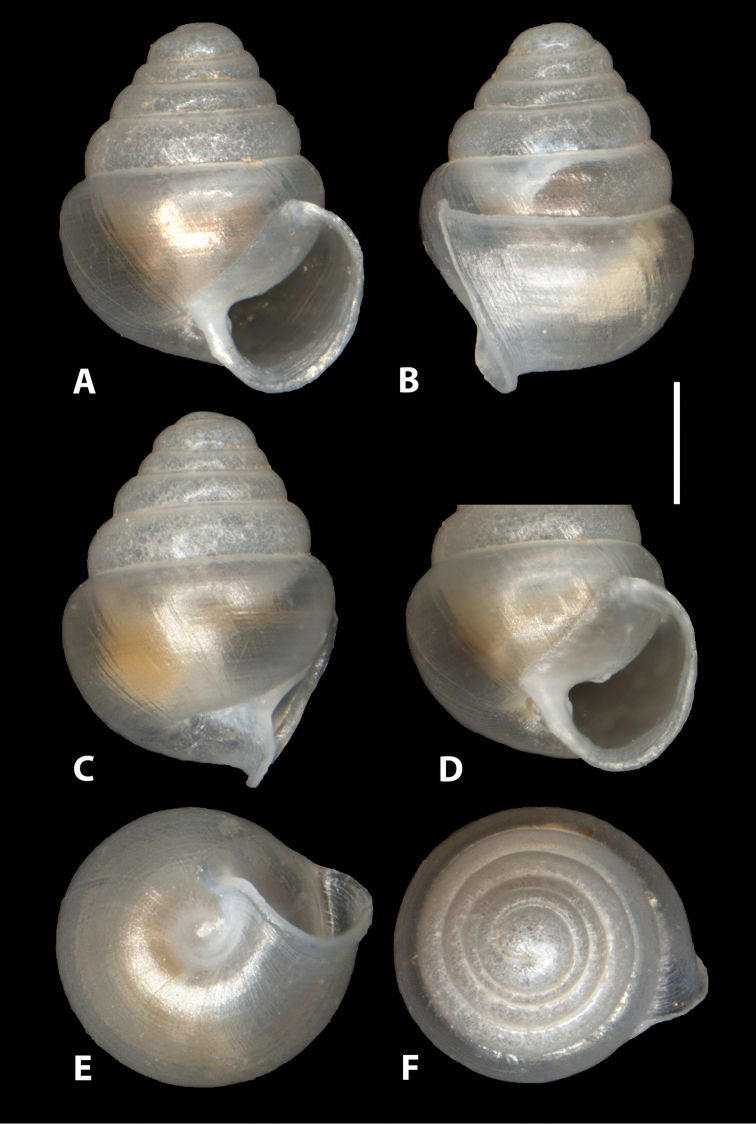
*Zospeum zaldivarae* sp. n., **A, E–F** different views of holotype shell (MNCN 15.05/60148H) **D** aperture in slightly oblique view showing apertural barriers.

**Figure 7.**
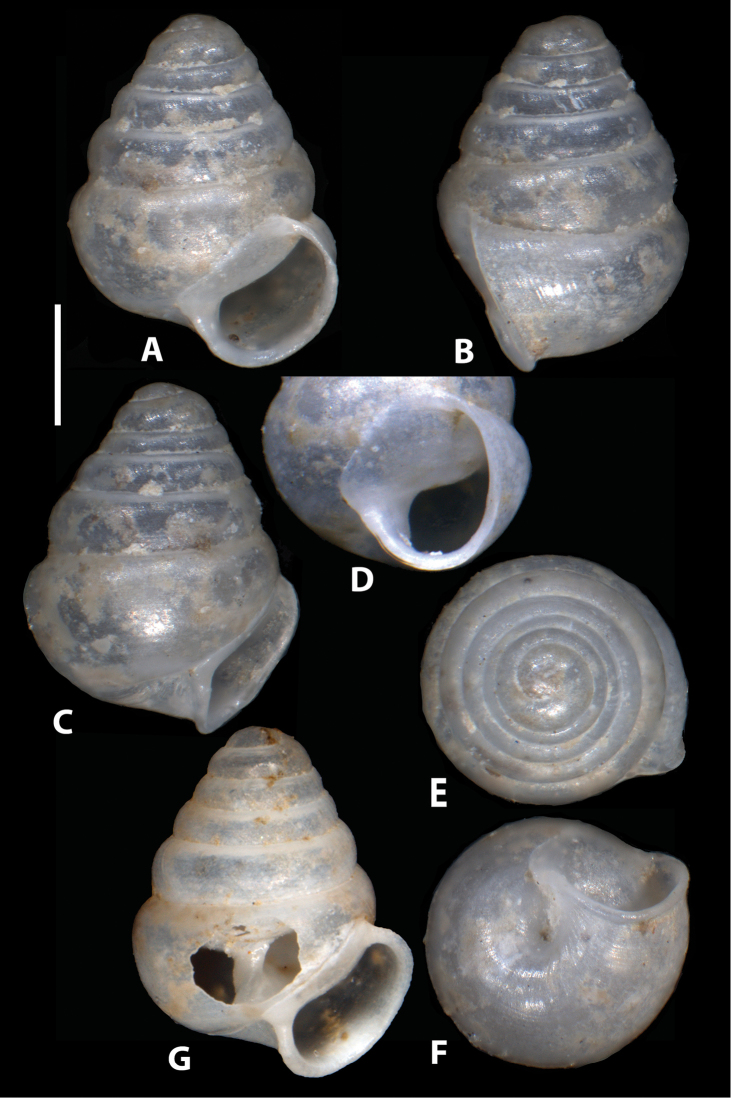
*Zospeum zaldivarae* sp. n., **A–F** different views of edentate paratype shell (RMNH.234152) **D** aperture in oblique view **G** paratype shell (UPV/EHU-FC: 72) with window in body whorl exposing columella.

Shell minute, turbinate-conical, with approximately 5 ½ regularly coiled, convex, rounded whorls; shell transparent when fresh, chalky white with age, comparatively large, rather variable in shape; teleoconch sculpture of irregular axial striae or blunt growth lines, often crossed by an equally superficial spiral element, some distinct axial ribbing may be present for a short distance immediately behind palatal-basal lip; last whorl large and tumid, encompassing *ca.* 2/3 of shell height; aperture lunate; peristome somewhat higher than wide, closely adhering to spire, taking up about half of the shell height, angular, with a thin, straight parietal callus; apertural dentition usually consisting of a small, short lamella on the parietal wall and a tooth on the parietal-columellar corner of the peristome (barely conspicuous in frontal view (Fig. 6A), best discernable in a slightly oblique, apertural view (Fig. 6D)); apertural barriers can however, be entirely absent (Fig. 7D); columella with a single, low, lamella-like dilatation (Fig. 7G), only visible in body whorl at some distance from the aperture.

##### Differential diagnosis.

Though comparatively large amongst Iberian species, the *Zospeum zaldivarae* shell is minute (shell height *ca.* 1.5 mm) and turbinate-conical in form. It is however, larger, wider (*ca.* 1.2 mm) and less elongate than other known Iberian *Zospeum* species except *Zospeum biscaiense*. *Zospeum biscaiense* has a more tightly coiled shell with palatal-basal apertural barriers.

##### Etymology.

The new species is named after Mª Pilar Zaldívar, a biologist and speleologist from the *Niphargus* Speleological Team, who discovered the species in the 1980’s.

##### Distribution.

Only known from the type locality.

##### Ecology.

**Figure 8.**
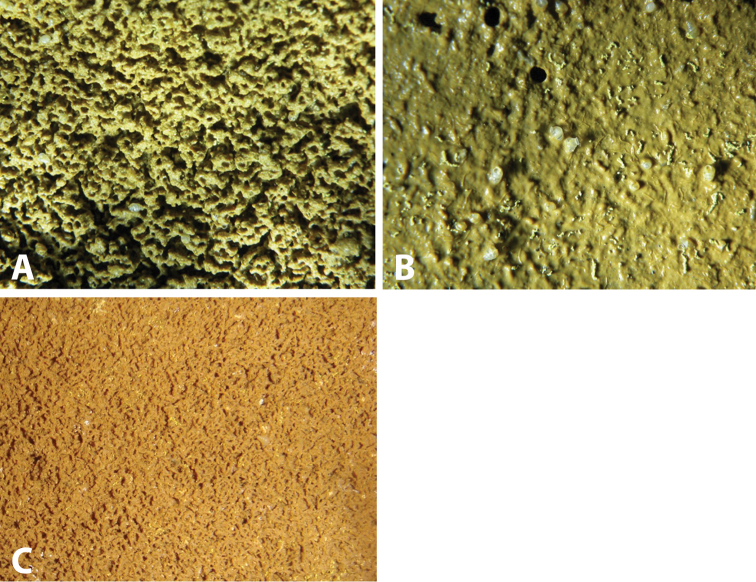
Habitat of *Zospeum* species. **A–B** Moist muddy layer with *Zospeum vasconicum* sp. n. in Cueva Arrikrutz; Prov. Gipuzkoa, Natural Park of Aizkorri-Araotz, Oñate **C** Muddy sediment matrix of *Zospeum zaldivarae* sp. n. habitat in Cueva de Las Paúles (locus typicus) with congener (*Zospeum suarezi*) in view.

*Zospeum zaldivarae* was found sparingly in a muddy sediment matrix of somewhat coarse, vermiform texture interspersed by clumped aggregations of yellow- and white-coloured fungi (Fig. 8C) (Jochum et al. 2012 fig. A–B). No bats or bat guano were seen in the vicinity of the collection site. The species was found syntopically with *Zospeum suarezi*.

##### Conservation.

*Zospeum zaldivarae* is only known from the Cueva de Las Paúles. Consequently, and in conjunction with the Guidelines for the IUCN Red List (IUCN Standards and Petitions Subcommittee 2014) it is a vulnerable, narrow range endemic (Vu D2) and as such, warrants immediate conservation priority. Although this cave belongs to the Natural Monument of Monte Santiago, it is nonetheless relatively easily accessible to the public.

##### Remarks.

*Zospeum zaldivarae* appears to be polymorphic in the presence/absence of apertural barriers. These barriers were not noticed in the material sequenced by Weigand et al. (2013), but their presence cannot be excluded. However, we have little doubt that the dentate and edentate specimens co-occurring at the type locality are conspecific.

*Zospeum zaldivarae* is conchologically quite different from most other Iberian *Zospeum* species hitherto described. In shape, it best resembles *Zospeum biscaiense*. These two species share a wide shell with a reniform aperture and an angular, not roundish, peristome with a straight palatal-columellar callus. Also, phylogenetically, this species is distinct (Weigand et al. 2013) and possibly more closely related to *Zospeum biscaiense*.

## Supplementary Material

XML Treatment for Zospeum vasconicum

XML Treatment for Zospeum zaldivarae
